# Understanding Khat: Its Sociocultural and Health Implications in Saudi Arabia

**DOI:** 10.7759/cureus.56657

**Published:** 2024-03-21

**Authors:** Mohammed D Al Shubbar

**Affiliations:** 1 College of Medicine, Imam Abdulrahman Bin Faisal University, Dammam, SAU

**Keywords:** recreational drugs, toxicology, illicit drugs, saudi arabia, health effects of khat, khat

## Abstract

This review offers an insight into the historical background, utilization, chemical composition, health impacts, processes, and cultural aspects associated with the usage of khat, a psychoactive, mind-altering plant indigenous to the Arabian Peninsula and the Horn of Africa. It further explores the cultural importance of khat in nations such as Saudi Arabia, Yemen, Ethiopia, and Somalia, detailing its chemical makeup containing alkaloids and other compounds, its physiological impact on the body, and its potential health risks like cardiovascular complications, mental health disorders, and dental problems. The cultural, economic, and religious aspects that affect perceptions and consumption of khat were also considered while emphasizing its usage despite legal bans in many nations.

## Introduction and background

Historical context of khat

Native to the Arabian Peninsula and the Horn of Africa, khat, or *Catha edulis*, is a flowering plant. It has a long history of usage in culture and customs, especially in southern Saudi Arabia, Yemen, Ethiopia, and Somalia. Chewing the plant's leaves, which contain psychoactive substances that have stimulant effects, is how khat is used [1].

Historical records reveal that khat was used for a variety of purposes in ancient civilizations, including religious rites, social gatherings, and therapeutic procedures. Its use goes back hundreds of years. Khat has become deeply embedded in the cultural fabric of the communities where it is farmed and consumed, playing an important part in social interactions and traditional events [1,2].

In recent times, the usage of khat has become widespread not only in its traditional growing regions but also in expatriate groups across the globe, including Saudi Arabia. Even though khat is outlawed in many countries, including Saudi Arabia, some people nevertheless use it, perhaps due to social acceptance and cultural norms in some regions and the stimulating effects they believe it to have. The leaves and buds of the khat plant can be either fresh or dried; there are forty-four varieties of khat. It is a shrub with green leaves that is primarily chewed; the flavor varies according to the amount of tannic acid in the leaves. The immature leaves are mildly sweet, whereas the mature leaves have a fragrant scent and an astringent flavor [2].

Given Saudi Arabia's stringent drug laws and a strong commitment to Islamic values, which forbid the use of intoxicating substances, khat can still be accessible in some areas, where it is sold and consumed by a particular age bracket, such as young men and foreign laborers, despite legal constraints and religious bans. This phenomenon could be attributed to the widespread belief among khat consumers that it enhances mental capacity and physical strength, alongside other factors particularly appealing to young males, such as improved sexual performance, heightened joy and climax, and its potential to aid in sleep [3].

## Review

Epidemiology

Chewing khat has long been considered a communal practice, especially among Ethiopian, Yemeni, and Somali communities. It is also used to counteract the effects of alcohol, lessen appetite and weariness, and improve attentiveness. Although khat leaves and buds are usually eaten raw, they may sometimes be converted into a drink by drying the plant leaves to be then used like tea. Most often, it is chewed for six to eight hours in social gatherings with eight to twenty individuals. Chewing on large quantities of khat (between 100 and 300 g) can take place during a three to four-hour session [2].

Traditional medicine used khat to treat respiratory conditions, stomach difficulties, respiratory disorders, cough, and influenza. Studies reveal the anti-bacterial, anti-cariogenic, and anti-oxidant qualities of khat leaves [4].

Research conducted in Jazan, Saudi Arabia, presented findings indicating that khat-related fatalities accounted for 3% of all deaths, a figure that escalated from 4% in 2020 to 9% in 2021. This comparison highlights a significant increase in the prevalence of khat-associated mortalities among the total number of fatal incidents recorded. The demographic profile of the individuals affected solely included males, with their ages spanning from 23 to 45 years. Analysis of postmortem samples revealed that 57% tested positive exclusively for khat, while the remaining 43% indicated the presence of khat in conjunction with other substances. The study meticulously quantified the average concentrations of cathinone and cathine, two active compounds in khat, finding levels of 85 ng/mL and 486 ng/mL in blood samples, respectively. These concentrations varied across different tissues, with 69 ng/mL and 682 ng/mL recorded in brain tissue, 64 ng/mL and 635 ng/mL in liver tissue, and 43 ng/mL and 758 ng/mL in kidney tissue. Furthermore, the blood concentrations for cathinone were noted to range between 18 to 218 ng/mL from the 10th to 90th percentiles, while cathine concentrations spanned from 222 to 843 ng/mL. The study underscores that a staggering 90% of khat-related deaths had cathinone levels exceeding 18 ng/mL and cathine levels surpassing 222 ng/mL. In cases where khat was the sole substance identified, homicide emerged as the predominant cause of death, representing 77% of such instances. This investigation sheds light on the alarming impact of khat consumption on mortality rates within the studied region, offering critical insights into the substance's pharmacological effects and its association with violent fatalities [5].

Recent investigations have revealed that 14.16% of university students have reported using khat, with the highest incidences observed in Jazan, a city in southern Saudi Arabia, and in Ethiopia. This study highlighted a pronounced gender disparity in khat consumption patterns, with male students demonstrating a significantly higher propensity for khat use compared to their female counterparts. The lifetime prevalence of khat use among the study population was found to be 27.31%, with male students engaging in khat chewing more frequently than female students. The findings from this survey further clarified that male students were more likely to have used khat in the past and continue to do so at present, indicating a gender-specific trend in the consumption of this substance [6].

Chemical composition

The physiological impacts of khat on the human body can be traced back to a diverse array of psychotropic compounds found within its leaves. These include a variety of alkaloids, terpenoids, sterols, flavonoids, glycosides, and tannins. Additionally, the leaves are rich in more than ten types of amino acids, such as tryptophan, glutamic acid, alanine, glycine, and threonine, which are crucial for protein synthesis and various metabolic processes. The nutritional content extends to trace amounts of essential vitamins like ascorbic acid (vitamin C), thiamine (vitamin B1), riboflavin (vitamin B2), niacin (vitamin B3), and carotene (a precursor to vitamin A). Furthermore, khat leaves contain significant levels of essential minerals and elements, including calcium, iron, manganese, zinc, and copper, which play vital roles in maintaining bodily functions and structural integrity. However, alongside these beneficial nutrients, khat leaves also contain toxic metals such as lead and cadmium, posing potential health risks. Moreover, a minimal amount of fluoride is present, which, in small doses, can be beneficial for dental health but may pose health concerns at higher exposures. The primary active ingredients contributing to khat's psychotropic effects are its alkaloids: cathinone, cathine, and nor-ephedrine. These compounds are central to the stimulant properties of khat, affecting the central nervous system and leading to both the desired and adverse effects associated with khat consumption [7].

The primary hallucinogenic ingredient in khat, cathinone, shares structural similarities with amphetamines. It stimulates the central nervous system, causing the brain to release more neurotransmitters like serotonin, norepinephrine, and dopamine. Feelings of euphoria, heightened awareness, and increased energy follow from this. Additionally, cathinone has the ability to reduce appetite, which could explain why those who use khat report feeling less hungry [4]. While not as strong as cathinone, the other alkaloid in khat, called cathine, produces comparable effects. It is regarded as a precursor to cathinone and is converted by the body into cathinone. Though its effects are not as strong as those of cathinone, cathine is nevertheless a stimulant. Moreover, similarly to cathinone, cathine can raise blood pressure, heart rate, and level of awareness [4,7].

Another substance found in khat that adds to its stimulant properties is nor-ephedrine. It shares structural similarities with the neurotransmitter norepinephrine, which is important in the body's reaction to stress. The sympathetic nervous system is triggered by nor-ephedrine, which raises blood pressure, heart rate, and metabolic rate. These effects add to khat's overall stimulant qualities and may provide feelings of excitement and enhanced energy [8].

Depending on a number of variables, including the dosage, frequency of usage, and individual susceptibility, consuming khat can have different physiological effects. Increased heart rate, raised blood pressure, dilated pupils, and decreased hunger are possible short-term consequences. Enhanced alertness, elevated mood, and euphoric sensations are also possible side effects for certain users. When using khat for an extended period, though, tolerance, dependence, and withdrawal symptoms can occur. Withdrawal symptoms are usually mild, and they commonly include depression, anxiety, nightmares and disturbance of sleep patterns, and an increase in appetite [7-8].

Health effects

Khat usage is associated with a number of unfavorable health outcomes despite its apparent benefits. Increased talkativeness, vitality, alertness, and heart rate are among the short-term impacts. Anxiety, impatience, and depression are comedown symptoms that linger for a whole day. Frequent use might result in mental health issues such as depression, psychosis, and aggressive conduct. Prolonged consequences encompass deteriorating mental well-being, difficulty with sleep, recurring hepatitis potentially leading to cirrhosis, infertility, gastrointestinal problems like esophagitis, gastritis and duodenal ulcers, and psychological reliance. Khat users are also at an increased risk of having some cardiovascular problems ranging from hypertension to myocardial infarction. Decreased blood pressure, fatigue, and mild depression are some of the signs of withdrawal [7].

Despite its widespread use, khat has some downsides. In addition to numerous detrimental health impacts, the stimulant qualities of khat can result in cardiovascular issues, psychological dependence, and gastrointestinal issues. Chronic khat use has also been linked to dental problems, anxiety, and insomnia [9]. However, case-control studies found that chewing khat is more common among those who are HIV-positive, particularly when paired with alcohol and unprotected sexual activity [4].

Ethiopian, Kenyan, and Yemeni khat trees are evergreens that contain more than 20 different chemicals, the primary active component of which is cathinone. The primary aim of the study conducted in Bahir Dar, Ethiopia, was to investigate how chewing khat affected blood pressure and oral health. Of the 422 male khat chewers in the study, 62% reported having dental health issues and having begun chewing khat at a young age. According to the study, people who chewed more than 100 grams in a single session had a 4.33-fold increased risk of dental health issues compared to people who chewed less frequently, who were 7.58 times more likely. Furthermore, there was a 7.25-fold increase in systolic blood pressure and a nine-fold increase in diastolic blood pressure in those who engaged in khat sessions lasting longer than six hours [10].

Consuming khat has broad societal and economic ramifications, extending beyond its physiological effects. Its use is widely accepted across various demographics, yet it imposes a significant economic burden, especially on lower-income households. Studies from Kenya, Somalia, and Ethiopia reveal that overindulgence in khat leads to lowered productivity, missed job or school opportunities, and strained relationships while also draining finances. In Kenya, the negative correlation between khat consumption and household economy is influenced by employment status, income level, and education. Similarly, in Somalia, the daily expenditure of $7.29 on khat signifies a considerable financial burden, potentially hindering the country's socioeconomic development. Furthermore, in Ethiopia, the financial strain is compounded by additional expenditures on alcohol and other commodities consumed alongside khat. This cycle of spending, often sustained through credit, exacerbates financial instability and social gaps, particularly affecting low-income individuals and families and deepening the cycle of poverty and social disparity [11].

Factors contributing to the use of khat

The usage of khat in Saudi Arabia is driven by a complex interaction of cultural, societal, religious, and economic elements that shape attitudes and views about the drug. In many cultures, chewing khat is an integral part of the social fabric and is frequently seen as a custom of historical importance. Chatting over khat at social events is a common way for people to interact and socialize, which helps explain why it's accepted by some demographic groups [12].

In Islam, the interpretation of khat's permissibility varies, with a significant number viewing it as haram (forbidden), while others consider it merely makruh (discouraged) or even halal (permissible). Contrary to the broader religious prohibition due to its implications for physical and mental health, a considerable portion of khat users do not regard its consumption as religiously banned. This is evident in certain nations, such as Yemen and Somalia. In Saudi Arabia, the Muftis, who are often referred to as scholars, unanimously concur that khat is prohibited [13]. Nonetheless, there exists a segment of khat consumers in Saudi Arabia who may hold differing opinions.

Economically, khat's accessibility and low cost are factors in its widespread usage, especially among those with lower incomes who could consider it to be a reasonably priced form of entertainment. The open sale of khat leaves in marketplaces and stores in Yemen has led to the commercialization of khat and the normalization of its usage in society, and as Yemen has borders to Saudi Arabia, the accessibility of khat in southern Saudi Arabia is expected to be high, relative to other parts of the country [14].

Mahfouz, in 2013, performed a study in Jazan, Saudi Arabia, to investigate how students, namely those in intermediate and high secondary schools, perceive the practice of chewing khat. Seventy to 80 percent of respondents agreed that it is a frequent behavior, according to the research. The study also showed that except for people living in Faifa mountain, where most of their khat users chew their own farmed khat, consuming khat significantly strains family finances. The majority of interviewees believed that chewing khat is forbidden in Islam (haram). Programs that modify behavior and promote social acceptance should be the main goals of prevention initiatives [15].

Public health concerns and future recommendations

To address public health issues associated with khat consumption in Saudi Arabia, a comprehensive approach is required. This involves carrying out public awareness campaigns aimed at urban and rural people, using different media platforms and instructional materials. It is essential to have a strong regulatory structure in place to manage the cultivation, sale, and consumption of khat. Community feedback is crucial for implementing a culturally responsive strategy. Rehabilitation programs are essential for addressing khat dependency by emphasizing abstinence and incorporating mental health care and counseling to disrupt the cycle of dependency. Researching socio-cultural aspects that influence khat usage can help create targeted interventions and assist policymakers in designing culturally suitable tactics. Developing strong surveillance systems is crucial for tracking patterns and evaluating the impact of actions. Saudi Arabia can effectively tackle the intricate issues related to khat consumption by integrating public awareness, regulation, rehabilitation, research, and surveillance to protect the health and well-being of its population. Saudi Arabia has recently launched an aggressive campaign against drugs, targeting all forms of narcotics and apprehending dealers. This initiative notably includes efforts against khat, representing a strategic and commendable move in the nation's battle against drug abuse. These efforts reflect a significant commitment to improving public health and safety.

Limitations

While this review provides a thorough and insightful examination of khat usage, covering historical, cultural, chemical, and health aspects, it has some minor limitations. The narrative synthesis approach, though effective in conveying the multifaceted nature of khat usage, may not capture the full statistical breadth of quantitative research, such as meta-analyses or systematic reviews. Additionally, the focus is predominantly on regions where khat is traditionally used, which might limit the generalizability of the findings to global contexts. However, these limitations do not detract from the overall value of the review. The article remains a significant contribution to the literature, offering a well-rounded perspective on the complexities of khat usage and its implications in the studied regions.

## Conclusions

In conclusion, while being prohibited and carrying some health hazards, khat is nonetheless a commonly used psychoactive drug in southern Saudi Arabia. Its widespread occurrence, especially in certain demographic groups, is a reflection of deeply embedded health problems. To address this public health issue and encourage better lives among Saudi citizens, efforts must be made to increase public knowledge of the harmful health consequences of khat and to enforce the laws that already forbid its use. To reduce the negative effects of khat use on public health and well-being, further research is required to better understand the trends and factors of khat consumption in Saudi Arabia and to design focused interventions.
